# Rare Development of Primary Parotid Gland Epithelial-Myoepithelial Carcinoma in a Child

**DOI:** 10.1155/2020/5837659

**Published:** 2020-06-30

**Authors:** Daniyah Saleh, Doaa Al Ghamdi

**Affiliations:** ^1^Department of Anatomic Pathology and Laboratory Medicine, King Faisal Specialist Hospital and Research Center, Jeddah, Saudi Arabia; ^2^Department of Anatomic Pathology and Laboratory Medicine, King Abdulaziz University Hospital, Jeddah, Saudi Arabia

## Abstract

Salivary gland tumors are uncommon in children. They consist of variable histopathological subtypes of benign and malignant tumors. EMC is a discrete entity among the WHO classification of salivary gland tumors since 1991. EMC is considered a low-grade malignant salivary gland tumor arising from intercalated ducts. Typically, it affects an adult female individual. Surgical resection with a negative margin is the mainstay treatment option. EMC has a potential for metastasis with a high rate of recurrence. Based on the available English literature, two cases of EMC diagnosed in a pediatric age group have been reported. Therefore, we describe the third EMC that developed in the left parotid gland of a young child. The diagnosis of EMC was established through histopathological examination of the total parotidectomy specimen and neck lymph node dissection, together with ancillary studies. Later, the patient suffered from cervical lymph node enlargement due to metastasis in which FNAB was taken. Metastasis from the known EMC was suspected with cytomorphological features in smears and cell block. Immunohistochemistry markers for the biphasic components were supportive of EMC. Due to advanced disease, the patient necessitated a concomitant treatment of radiochemotherapy. Besides, there was radiological evidence of bilateral multiple lung metastatic nodules. However, a biopsy was not sent for pathological confirmation.

## 1. Introduction

Epithelial-myoepithelial carcinoma (EMC) is a rare low-grade biphasic (epithelial and myoepithelial population) malignant salivary gland tumor [1]. It accounts for 0.5% among salivary gland tumors with a female predilection aged between 60 and 70 years old [1]. The parotid gland is the most common site of involvement [1]. EMC has a significant rate of local recurrence representing 42% [2]. There are 10% of cases with periparotid and cervical lymph node metastasis documented [2]. Complete surgical removal either by total or subtotal parotidectomy is the treatment of choice for EMC [2]. As per English literature, although EMC is a tumor of an adult, there are seldom reported cases of pediatric EMC of parotid gland limited only to two cases [3, 4]. Hence, from this point of view and due to the rarity of EMC in the pediatric population, we describe the third case of EMC that developed in the parotid gland of a young child diagnosed through histopathological examination and helpful Immunohistochemistry profile for both neoplastic cell components. Subsequently, recurrence has occurred, cervical lymphadenopathy was positive for metastasis, and there was imaging-based evidence of lung metastasis.

### 1.1. Case Presentation

**Figure 1:**
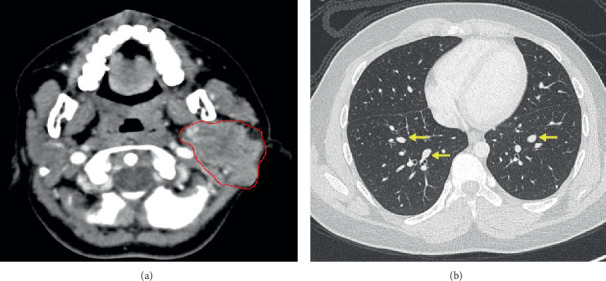
(a) Axial plane of CT neck soft tissue with contrast shows a large heterogenous density lesion involving the left parotid gland (*red irregular circle*) and extending into the overlying subcutaneous tissue. (b) CT chest with contrast, axial plane; bilateral scattered lung nodules of variable size range 2-13 mm (yellow arrows).

**Figure 2:**
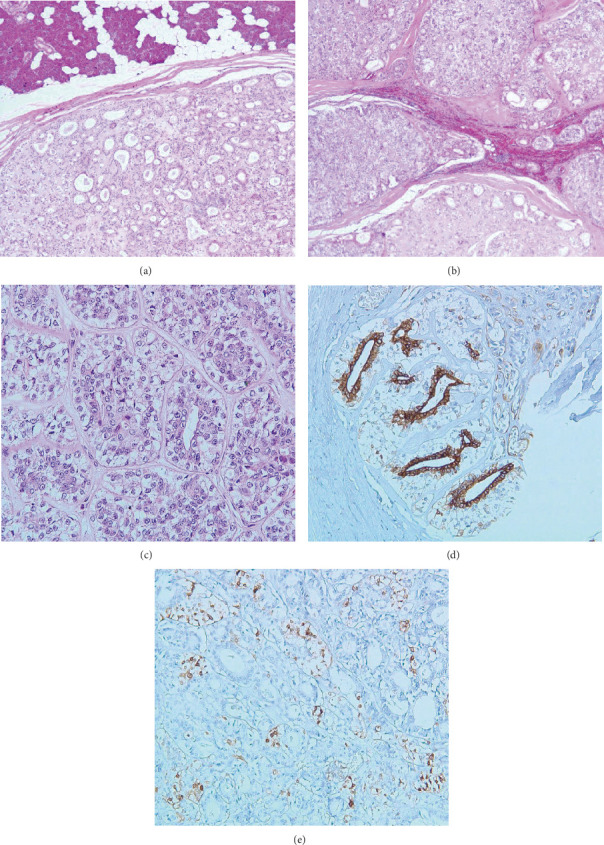
(a) H&E, 4x; an encapsulated tumor with adjacent normal serous parotid gland parenchyma. (b) H&E, 4x; multinodular pattern of the tumor separated by delicate fibrovascular septa. (c) H&E, 20x; cytomorphological features of the dual types of tumor cells with inner ductal epithelial layer and outer neoplastic myoepithelial cells having a clear cytoplasm due to glycogen content. No obvious cytological atypia or necrosis. Scarce mitotic figure. (d) The inner ductal epithelium express cytokeratin 5/6. (e) The outer myoepithelial cells express S-100.

**Figure 3:**
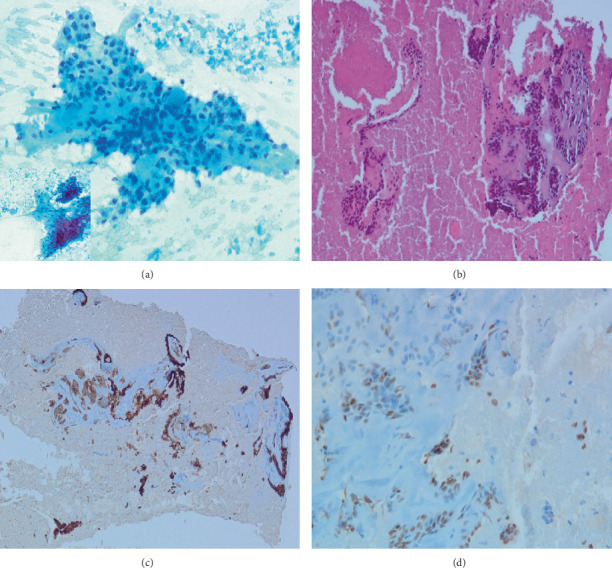
(a) FNAB of the left supraclavicular lymph node reveals syncytial clusters of pleomorphic cells exhibiting high N/C ratio, nuclear hyperchromasia, and prominent nucleoli. (b) Cell block of the previous pleomorphic clusters with abundant necrotic background. (c) Cytokeratin 7 immunohistochemical stains the inner ductal epithelium. (d) The outer myoepithelial cells are highlighted by P63.

A 13-year-old boy not known to have any medical illnesses presented to King Abdul-Aziz University Hospital (KAAUH) complaining of progressive, painless left facial swelling accompanied by facial nerve palsy since few months before admission. By palpation, the left facial swelling was a solid mass in nature, irregular in shape, and adherent to deep soft tissue. A computerized tomography (CT) scan of the neck and soft tissue was performed and found to have a large heterogeneous soft tissue density involving both superficial and deep portions of the left parotid gland associated with multiple foci of calcification (Figure 1(a)). The lesion was extended into the subcutaneous fatty tissue and bulged into the overlying skin. It measures about 6 × 4.6 × 4.5 cm at its maximum craniocaudal, anterior-posterior, and transverse dimensions, respectively. It is encroaching on the adjacent part of the ipsilateral sternomastoid muscle. He underwent total left parotidectomy with left neck dissection. A portion of the facial nerve was sent for intraoperative frozen section consultation and it was uninvolved by the tumor. Gross specimen of the parotid gland depicts an ill-defined, gray-white, firm lobulated mass (7 × 4 × 3.8 cm) with scattered microcystic-like spaces that are seen throughout the cut sections. The mass is almost reaching the surgical resection margin. Microscopic examination revealed a multinodular neoplastic growth rimmed by a partial thick fibrous capsule. The neoplastic nodules are separated by delicate fibrovascular septa and comprise the dual-cell population of inner epithelial cells surrounded by outer myoepithelial cells (Figures 2(a) and 2(b)). The majority of the tumor cells have myoepithelial features with clear cytoplasm or naked nuclei (Figure 2(c)). Mild nuclear pleomorphism and infrequent mitotic figures were noted. Lymphovascular invasion is identified but there was no perineural invasion. Immunohistochemistry markers showed diffuse positivity of cytokeratin 5/6 in the epithelial (ductal) component (Figure 2(d)) and immunoreactivity for the S-100 stain in the myoepithelial component (Figure 2(e)). A total of 86 lymph nodes were examined and all were negative for metastasis. Based on the classic morphology, immunohistochemistry profile supports the diagnosis of epithelial-myoepithelial carcinoma. At postresection, six cycles of chemotherapy (docetaxel) of 3-week intervals between each cycle in concomitant with 60 Gy radiotherapy were given. Two years later, a follow-up evaluation of the CT chest imaging reveals bilateral scattered lung nodules suggestive of lung metastasis (Figure 1(b)). However, no specimen was received for the microscopic confirmation of the lung metastasis. On the other hand, follow-up evaluation of the CT neck soft tissue imaging study visualizes a left neck heterogenous enhancing mass (3 × 2.3 × 1.8 cm) suggestive of the recurrence of the malignant tumor. Also, there is a mild increase in the size of the left supraclavicular lymph node (2.9 × 2.4 cm), in which fine-needle aspiration (FNA) was obtained. Microscopic evaluation of the smears and cell block reveals syncytial clusters of pleomorphic cells exhibiting increased nuclear to cytoplasmic ratio, hyperchromatic nuclei, and prominent nucleoli. Abundant necrosis in the background (Figure 3(a)) was found. Immunophenotype of those pleomorphic cells is similar to the known left parotid epithelial-myoepithelial carcinoma (Figures 3(b)–3(d)).

## 2. Discussion

Head and neck EMC is a rare low-grade neoplasm, representing less than 1% of salivary gland tumors [1]. Donath et al. was the first author who applied the term EMC in 1972 [5]. Moreover, EMC has been recognized as a discrete entity in the WHO classification of salivary gland neoplasms since 1991 [6]. The vast majority of cases have been found to develop in the parotid gland. Although elderly females are more affected usually in their 7^th^ decade of life, two reported EMC cases have been described in the parotid gland of a young child followed by recurrence and lung metastasis after three years [3]. Similarly, our case illustrates the EMC of the parotid gland traced by recurrence, cervical lymph node metastasis, and lung metastasis in an approximately two-year duration posttotal parotidectomy and neck dissection. EMC has a nonspecific clinical presentation as it is a slow, painless growing mass; however, it is uncommon to be accompanied by facial palsy [7]. One study has hypothesized that EMC originated from intercalated ducts; however, the exact origin remains unclear [2]. Microscopically, EMC is typically composed of a dual population of cells arranged in a glandular/tubular growth pattern. The glands/tubules are lined by ductal epithelium encircled by neoplastic myoepithelial cells with clear cytoplasm. Very little cellular atypia and infrequent mitotic figures are usually noticed. Based on the hematoxylin and eosin (H&E) cytomorphological features of this neoplasm, our differential diagnosis includes myoepithelioma, myoepithelial carcinoma, and adenoid cystic carcinoma. A feature against myoepithelioma and myoepithelial carcinoma is the presence of inner ductal epithelial cells in EMC. On the one hand, adenoid cystic carcinoma is a biphasic tumor consisting of epithelial and myoepithelial cells similar to EMC components; however, these cells are smaller and usually have more hyperchromatic, irregular, and angulated nuclei which are not seen in EMC. Furthermore, immunohistochemical (IHC) markers help distinguish EMC from other salivary gland tumors. Typically, normal and neoplastic myoepithelial cells express S-100, smooth muscle actin, P63, and other myoepithelial markers. Besides, the ductal epithelial cells are positive for cytokeratin and EMA. The myoepithelial cells are PAS-diastase sensitive due to glycogenated cytoplasm. Using fine-needle aspiration biopsy (FNAB) to differentiate between the abovementioned salivary gland tumors is quite difficult and inaccurate as they may have overlapping cytological features such as the presence of extracellular material and clusters of epithelial and myoepithelial cells [8]. Since EMC is known to have a high recurrence rate and a metastatic potential, the tumor may behave aggressively. It is characterized by a solid growth pattern, necrosis, DNA aneuploidy, nuclear atypia, high mitosis, and involvement of the resection margin [9]. Adequate resection with free margin is the recommended therapeutic modality of low-grade EMC [9]. On the other hand, for cases with positive lymph nodes, neck dissection in concurrent with chemotherapy and radiotherapy are necessary. Until now, there are no studies of these treatment options [7].

## 3. Conclusion

Despite EMC having female predominance with the peak incidence in the sixth and seventh decade of life, it has been described in young children and male individuals in many studies. A high index of suspension is valuable, especially when evaluating a parotid gland mass in children. Careful histopathological examination for features defining the aggressive behavior of EMC is important. Patients with advanced disease and nodal involvement need more aggressive therapy. Close periodic follow-up is necessary since EMC has an increased rate of recurrence and has a potential for metastasis.

## Data Availability

Not declared.
